# Tragedies of War Bread

**Published:** 1917-12-08

**Authors:** 


### Tragedies of War Bread.

BY A STUDENT OF THOMAS LOVE PEACOCK'S SCHOOL.

Reports of domestic tragedies resulting from the use and abuse of war bread continue to flow in. Up to the time of writing no commission has been appointed to inquire into the following cases :

1. Dangers of Giving Them Rope.—A curious case is reported from North Exshire. John Deadhead, an ex-cinema actor, had for some time suffered from indigestion and depression. He had, it appears, found it increasingly hard to pay his debts owing to a morbid dread of parting with money. On Tuesday evening last he appeared much more cheerful, and chatted with his friends of the large quantity of "rope" in the bread. On Wednesday morning his landlady was horrified to find his dead body dangling from the gas-bracket. He had hanged himself with four feet of war-bread rope.

2. Death from Misadventure.—A sad case is on record of an old couple living in the workhouse at Y. They were from all accounts a most devoted pair, and assured the chaplain they had never quarrelled since the day they were married—and not very much before. On Wednesday evening last Granfer Smith (they were known respectively as "Granfer" and "Grandma") playfully threw a loaf of war bread at Grandma. She collapsed, and died of acute indigestion a few hours afterwards.

3. Murder or Manslaughter?—From Little Fontleroy comes the story of a poor old widow woman living by herself in a two-roomed cottage. Last week an old tramp (known locally as "Weary Willie") called at her door and explained that he hadn't had a crust since the war began. The poor old soul rushed into the cottage and brought him out a slice of war bread. Willie swallowed it whole, and died two hours afterwards. The widow will have to stand her trial.

4. Bread Mightier than Cheese.—An eminent scientist of X, while experimenting with a loaf of war bread in his laboratory, encountered a unique case of "frightfulness." For five days he had been endeavouring to determine the constituents of a cottage loaf. On the laboratory table was a piece of Dutch cheese, also awaiting analysis. He was called away to the telephone for a few minutes, and on his return the cheese had completely disappeared. Subsequent investig tion left no doubt whatever that the bread mites had eaten the cheese mites.

